# Optimized sliding mode controller for trajectory tracking of flexible joints three-link manipulator with noise in input and output

**DOI:** 10.1038/s41598-023-38855-7

**Published:** 2023-08-02

**Authors:** Muhammad I. Azeez, A. M. M. Abdelhaleem, S. Elnaggar, Kamal A. F. Moustafa, Khaled R. Atia

**Affiliations:** 1 Mechanical Design and Production Engineering Department, Zagazig University, Zagazig, 44519 Egypt; 2 Industrial Engineering Department, Zagazig University, Zagazig, 44519 Egypt

**Keywords:** Electrical and electronic engineering, Mechanical engineering

## Abstract

The aim of this study is to enhance the performance of a nonlinear three-rigid-link maneuver (RLM) in terms of trajectory tracking, disturbance and noise cancellation, and adaptability to joint flexibility. To achieve this, an optimized sliding mode controller with a proportional integral derivative surface (SMC-PID) is employed for maneuver control. An improved artificial bee colony algorithm with multi-elite guidance (MGABC) is utilized to obtain optimal values for the sliding surface and switching mode gain and attain the best performance for the robot maneuver system. The selection of the MGABC algorithm is based on its efficient exploration and exploitation techniques. The performance of the optimized SMC-PID robotic system is compared against other optimization algorithms found in existing literature, including Particle Swarm Optimization (PSO), Genetic Algorithm (GA), Artificial Bee Colony (ABC), Ant Lion Optimizer (ALO), and Grey Wolf Optimizer (GWO). The implemented controller effectively reduces the tracking error to 0.00691 radians, eliminates chattering phenomena in the control effort, and demonstrates robustness against disturbances and noise. The controller ensures that the objective function (OBJF) is minimized, with 0.954% increase in OBJF under low disturbance and noise conditions and 14.55% under severe disturbance and noise conditions. Moreover, the optimized controller exhibits resilience to variations in payload mass analysis, with the percentage increase in OBJF values ranging from 5.726% under low uncertainty conditions to 18.887% under severe uncertainty conditions. Flexible-link maneuvers (FLM) offer advantages such as improved safety and increased operating speeds in real-world applications. In this study, we investigated the impact of joint flexibility on the performance of the FLM system. Our proposed controller demonstrated superior tracking performance, characterized by minimal vibrations in the movement of the end effector.

## Introduction

Robotic maneuvers have gained significant prominence in the contemporary industrial landscape, contributing to improved product quality, enhanced productivity, increased accuracy, heightened speed, and enhanced flexibility within work environments. These maneuvers are increasingly employed in hazardous, monotonous, or repetitive industrial processes, despite the complex and demanding nature of industrial applications. Material picking and placing operations are among the fundamental applications of mechanical robotic maneuvers. Furthermore, industrial maneuvers find utility in welding, assembling, manufacturing, painting, and various operations within the automotive industry, as well as in the handling of radioactive and biohazardous materials during robotically assisted surgery. The imperative for enhanced efficiency and high-quality manufacturing has driven the pursuit of more robust and sophisticated robotic maneuvers^1–4^.

The mathematical modeling is known to present considerable difficulties and time constraints, especially when dealing with complex systems featuring strong interdependencies and multiple inputs and outputs. Recent studies have conducted a thorough and comprehensive examination of diverse mathematical modeling approaches, aiming to shed light on their strengths and limitations^2^. Moreover, a new simulation technique for physical modeling was compared to traditional mathematical methods for dynamic modeling of multi-DOF robotic manipulators, offering valuable insights into the distinctive mathematical characteristics associated with the implementation of Simscape software^1,5–9^.

Simscape Multibody models, also known as SimMechanics models, have gained popularity due to their ease of access, simulation capabilities, and control feasibility^1,8^. The utilization of simulation software provides several advantages, such as gaining insights into the behavior of multi-degree-of-freedom (DOF) robotic maneuver systems in a simulated environment and circumventing the complexities associated with mathematical formulations. Additionally, SimMechanics offers valuable tools for analyzing the flexibility of joints and links, which can be challenging to achieve using traditional mathematical derivation methods. However, a literature review reveals that this specific area requires further attention, as limited research has been conducted in this domain.

Alandoli et al.^1^ investigated the modeling and control of Flexible Link Manipulators (FLMs) using the Finite Element Method (FEM), and the dynamic equation was derived using Euler–Lagrange’s equation. To validate the accuracy of the mathematical model, a Simscape model was developed, and the open-loop responses of both models were observed to exhibit similar and concurrent increases. This validation process confirms the reliability of the Simscape model for the FLM system.

The validation of the system modeling was performed by designing a 3DOF rigid-link articulated manipulator in Simscape and comparing it with the analytically modeled system^7^. The outputs obtained from both simulations exhibited complete similarity, indicating a high level of agreement between the Simscape model and the analytically modeled system.

The primary goal of this study^10^ was directed towards tackling the obstacles linked to dynamic modeling. of a 7-DOF hydraulically actuated tele-operated robotic manipulator. The objective was to create a robust simulator capable of accurately deriving the arm’s dynamic and kinematic characteristics. To achieve this, a numerical model was developed using Simulink, incorporating the SimMechanics and Simscape toolboxes. Notably, the model accounted for uncertain parameters, allowing for a more comprehensive and realistic representation of the system's behavior.

In this study^6^, the primary emphasis was on utilizing Sim-Mechanics software as a valuable tool for modeling multi-DOF robotic manipulators in a manner that does not require explicit mathematical equations or relations. The Sim-Mechanics block model was employed to analyze variations in reaction forces and reaction torques acting on both 2DOF and 3DOF planar robotic manipulators.

Controlling robot manipulators is an intriguing field of study due to the complex dynamics involved. The dynamical analysis of robotic models aims to understand the relationship between the positions of the robotic arm and the joint torques applied by the actuators. Achieving accurate and reliable control is challenging due to the inherent coupling relations and nonlinear dynamics of the system. Conventional control techniques that rely on the dynamics of the robotic system struggle to address model uncertainties, including dynamic parameters (such as inertia and payload conditions), dynamic effects (such as complex nonlinear frictions), and unmodeled dynamics^11,12^.

The sliding mode control (SMC) technique has gained significant popularity in uncertain systems due to its numerous benefits, including robustness, order reduction, ease of implementation, strong resistance to external disturbances, low sensitivity to changes in system parameters, and design simplicity. To apply SMC design, a sliding surface must first be established to ensure the desired convergence property. Subsequently, an SMC is constructed to guide the system states towards the selected position, unaffected by uncertainty or disturbances. The underlying concept of SMC is to maintain the control signal on the sliding surface by inducing it to move in that direction^11,13–16^.

However, the discontinuous nature of the SMC law can lead to a chattering effect, characterized by high-frequency oscillations of the controlled variable when the system state reaches the sliding surface. This chattering effect can have detrimental consequences on actuator control and excite unwanted unmodeled dynamics^13,14^.

Recent research has been dedicated to the investigation of proportional integral derivative sliding mode controllers (SMC-PIDs) as robust nonlinear controllers. In the context of a three-phase active power filter, an adaptive dynamic global sliding mode controller with a proportional integral derivative (PID) sliding surface has been employed. This controller aims to achieve overall robustness, minimize steady-state error, and enhance system response speed^17^.

To optimize the control parameters of the fuzzy controller in the Adaptive PID Fuzzy Sliding Mode Control (APIDFSMC) for handling uncertain systems, a GA-based adaptive PID fuzzy sliding mode control strategy (APIDFSMC-GA) has been introduced^18^.

For uncertain nonlinear systems, an adaptive robust controller has been developed using a proportional-integral-derivative-type nonsingular fast terminal sliding mode control. This controller aims to provide essential characteristics such as rapid transient response, finite-time convergence, negligible steady-state error, and chattering cancellation^19^.

In the control of a nonlinear 2-DOF system using a sliding mode controller (SMC) with a PID surface, various optimization techniques have been explored. These techniques include the Antlion Optimization Algorithm (ALO), Sine Cosine Algorithm (SCA), Grey Wolf Optimizer (GWO), and Whale Optimizer Algorithm (WOA)^14^.

The selection of an optimal sliding surface and switching mode controller’s gain is a crucial aspect in enhancing the performance and efficiency of a sliding mode controller (SMC) with a proportional integral derivative (PID) surface. Traditional optimization methods often fail to deliver satisfactory results when optimizing these parameters. In the realm of scientific research and technical applications, there has been a notable surge in the utilization of evolutionary algorithms (EAs) as efficient and effective optimization approaches. This rise in popularity is primarily due to the fact that modern practical optimization problems tend to be non-convex, discontinuous, or even non-differentiable, posing significant challenges for traditional optimization techniques such as gradient-based approaches^20–23^.

Swarm-based optimization algorithms (SOAs) employ natural processes to guide the search for optimal solutions. Unlike simple optimization algorithms such as stochastic process and hill climbing, SOAs utilize a population of solutions instead of a single solution per iteration. This distinction is significant, as a population of solutions is processed and created at each iteration^24^.

Evolutionary algorithms encompass various unique paradigms, including Jellyfish Search Optimization (JSO), Glowworm Swarm Optimization (GSO), White Whale Optimization (WHO), Particle Swarm Optimization (HSO), Grey Wolf Optimization (GWO), Genetic Algorithm (GA), and Artificial Bee Colony (ABC) algorithm.

Among these, the Artificial Bee Colony (ABC) algorithm stands out as a relatively recent swarm intelligence-based optimization paradigm, known for its simplicity and effective performance. The basic ABC approach, initially proposed by Karaboga^20^, mimics the foraging behavior of a bee colony to maximize honey collection from the search space and identify the optimal food source. The ABC algorithm has exhibited superior performance compared to other well-known evolutionary algorithms, including FA, ACO, and CSO, across various common objective functions^25–28^.

The ABC algorithm comprises three types of bees: employed, onlooker, and scouting bees. Employed bees search for food at specific locations, gather it, and return to the hive. They perform a waggle dance, which conveys information about the location, proximity, and fitness of a flower patch, enabling efficient communication within the colony. This information facilitates the selection of promising flower patches without the need for external guides^24,29^. Food supplies are distributed among neighbors in each iteration, generating novel solutions that are evaluated using the fitness function. Follower bees are dispatched to more promising regions, while abandoned food sources are replaced. This enables the colony to collect food swiftly and effectively^20,30^. However, like other evolutionary algorithms, the ABC algorithm faces challenges related to early convergence and slow convergence rates when tackling complex optimization problems^23,26,31^. The exploration technique equation in the basic ABC algorithm performs well in searching for new food sources but exhibits limitations during exploitation, as highlighted by various studies in the ABC community^28,32,33^. Consequently, there has been significant focus on maximizing exploitation while enhancing the performance of the ABC algorithm. Balancing exploitation and exploration is crucial for evolutionary algorithms, as these two aspects are inherently antagonistic. Achieving a proper balance between the two is challenging but essential for boosting the performance of the ABC algorithm^23,26,31^.

The MGABC algorithm refers to an enhanced version of the Artificial Bee Colony (ABC) algorithm that incorporates multi-elite guidance to improve its performance^28^. The enhancements introduced in MGABC involve two key modifications. Firstly, the neighborhood search method has been enhanced to improve the search process within the algorithm. Secondly, two new food search methods have been developed for the exploration and exploitation stages of the algorithm. These methods rely on a selected set of superior food sources, known as the elite solutions. The inclusion of this group of elite solutions aims to maximize exploitation while ensuring that exploration is not compromised, leveraging the available information to strike a balance between the two objectives.

The present study focuses on utilizing the Lagrangian method and the Simscape multibody toolbox in MATLAB to develop a model for a 3-DOF rigid link maneuver and investigate the effectiveness of the proposed model in a representative multi-degree-of-freedom system, while keeping the complexity manageable for a comprehensive analysis. In order to validate the model, the open-loop system responses of both the mathematical and Simscape models are examined in section “Modelling the three-link robotic maneuver”. The proposed approach of using a sliding mode controller (SMC) with a proportional integral derivative (PID) sliding surface is presented in section “Sliding mode controller with PID surface”. Section “Metaheuristic optimization algorithm” elaborates on the enhanced artificial bee colony with multi-elite guidance (MGABC) metaheuristic optimization technique employed in the study. The optimization process using MGABC to enhance the overall robustness, stability, and tracking performance of the robotic system is discussed in section “Controller optimization for trajectory tracking”. A comprehensive performance analysis is conducted in section “Detailed simulated performance evaluation” to evaluate the proposed controller’s capability in mitigating disturbances and noise, as well as its robustness to variations in payload mass. Additionally, the adaptability of the controller to the flexibility of the three joints of the maneuver is examined. Finally, section “Conclusion” provides a summary of the concluding remarks of the study.

Throughout the entire study, bold lowercase letters are used to represent vectors, while uppercase letters are used for matrices.

## Modelling the three-link robotic maneuver

The decision to select a 3 DOF robotic maneuver for our study was driven by the objective of examining and showcasing the efficacy of the proposed model in a representative multi-DOF system. This choice was made while considering the need to maintain a manageable level of complexity for a comprehensive analysis. It is important to note that the first 3 angles of a robotic manipulator are often considered the most critical DOFs in industrial robots. This is because the remaining DOFs are typically incorporated in the end-effector, which generally exhibits less dynamics complexity compared to the base structure of the manipulator.

The purpose of the modeling is to develop a mathematical representation of the system that accurately describes its behavior and dynamics. The model will be derived using established mathematical methods and principles, such as the Lagrangian method, which considers the kinetic and potential energies of the links. The model will be designed to capture the complexity of the system and its interactions, incorporating various variables and assumptions, as necessary.

As the complexity of a problem increases, the need for variables, assumptions, and iterations also increases, resulting in prolonged computing times. However, Simscape Multibody, which is seamlessly integrated with MATLAB and Simulink, offers a potent solution for rapidly modeling, simulating, and analyzing systems. The tool is capable of accurately simulating both processes and systems, allowing for the analysis and prediction of system behavior. The model is constructed by arranging blocks from the Simulink library on the diagram canvas, endowing Simulink with an inherent versatility that makes it suitable for a wide range of applications. Additionally, Simulink serves as a valuable tool for testing and verifying models, enabling the simulation of a model’s behavior under diverse conditions. This functionality can be exploited to evaluate the limits of a model and locate potential errors. The subsequent subsections will also encompass the Simscape modeling and validation of the mathematical model using the Simscape model.

### Mathematical modelling

**Figure 1:**
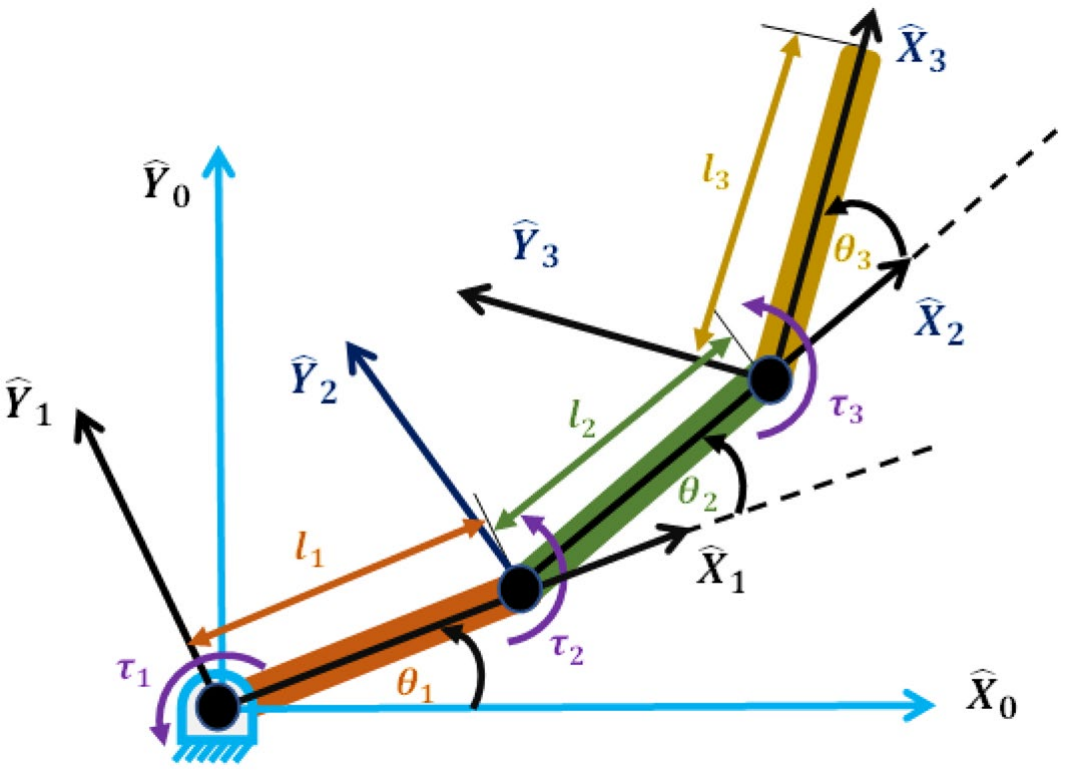
The three DOF robotic maneuver model.

Let us consider the three rigid-link planar robotic maneuver model shown in Fig. 1 and the mathematical model of the robotic maneuver with three rotating rigid links can be described by a second order nonlinear ordinary differential equations as shown in Eq. (1) by applying Lagrange’s principle^3,12,34^.

In the given system, ${\varvec{\theta}}\left({\varvec{t}}\right),\dot{{\varvec{\theta}}}\left({\varvec{t}}\right),\mathrm{and} \; \ddot{{\varvec{\theta}}}({\varvec{t}})$ represent the vectors of joint angles, joint velocities, and joint accelerations, respectively, for the three links. The 3 × 3 matrix represents the mass inertia matrix, which is symmetric and positive definite. ${\varvec{C}}\left({\varvec{\theta}},\dot{{\varvec{\theta}}}\right)$ and ${\varvec{G}}\left({\varvec{\theta}}\right)$ represent the vectors of centrifugal and Coriolis forces, and gravitational forces, respectively. Additionally, ${\varvec{\tau}}({\varvec{t}})$ represents the vectors of torques applied at each joint of the three links.

1 $$\left[ {\begin{array}{*{20}c} {m_{11} } & {m_{12} } & {m_{13} } \\ {m_{21} } & {m_{22} } & {m_{23} } \\ {m_{31} } & {m_{32} } & {m_{33} } \\ \end{array} } \right]\left[ {\begin{array}{*{20}c} {\ddot{\theta }_{1} } \\ {\ddot{\theta }_{2} } \\ {\ddot{\theta }_{3} } \\ \end{array} } \right] + \left[ {\begin{array}{*{20}c} {C_{1} \left( {\theta ,\dot{\theta }} \right)} \\ {C_{2} \left( {\theta ,\dot{\theta }} \right)} \\ {C_{3} \left( {\theta ,\dot{\theta }} \right)} \\ \end{array} } \right] + \left[ {\begin{array}{*{20}c} {G_{1} \left( \theta \right)} \\ {G_{2} \left( \theta \right)} \\ {G_{3} \left( \theta \right)} \\ \end{array} } \right] = \left[ {\begin{array}{*{20}c} {\tau_{1} \left( t \right)} \\ {\tau_{2} \left( t \right)} \\ {\tau_{3} \left( t \right)} \\ \end{array} } \right]$$

where

2 $$\begin{aligned} m_{11} & = l_{1}^{2} \left( {0.25m_{1} + m_{2} + m_{3} } \right) + l_{2}^{2} \left( {0.25m_{2} + m_{3} } \right) + 0.25l_{3}^{2} m_{3} + l_{1} l_{2} \; cos\left( {\theta_{2} } \right)\left( {m_{2} + 2m_{3} } \right) \\ & \;\;\; + l_{2} l_{3} m_{3} \; cos\left( {\theta_{3} } \right) + l_{1} l_{3} m_{3} \; cos\left( {\theta_{2} + \theta_{3} } \right) + I_{1} + I_{2} + I_{3} \\ \end{aligned}$$

3 $$\begin{aligned} m_{12} & = l_{2}^{2} \left( {0.25m_{2} + m_{3} } \right) + 0.25l_{3}^{2} m_{3} + l_{1} l_{2} \; cos\left( {\theta_{2} } \right)\left( {m_{3} + 0.5m_{2} } \right) + l_{2} l_{3} m_{3} \; cos\left( {\theta_{3} } \right) \\ & \;\;\; + 0.5 l_{1} l_{3} m_{3} \; cos\left( {\theta_{2} + \theta_{3} } \right) + I_{2} + I_{3} \\ \end{aligned}$$

4 $$m_{13} = 0.25l_{3}^{2} m_{3} + 0.5l_{2} l_{3} m_{3} \; cos\left( {\theta_{3} } \right) + 0.5l_{1} l_{3} m_{3} \; cos\left( {\theta_{2} + \theta_{3} } \right) + I_{3}$$

5 $$\begin{aligned} m_{21} & = l_{2}^{2} \left( {0.25m_{2} + m_{3} } \right) + 0.25l_{3}^{2} m_{3} + l_{1} l_{2} \; cos\left( {\theta_{2} } \right)\left( {m_{3} + 0.5m_{2} } \right) + l_{2} l_{3} m_{3} \; cos\left( {\theta_{3} } \right) \\ & \;\; + 0.5l_{1} l_{3} m_{3} \; cos\left( {\theta_{2} + \theta_{3} } \right) + I_{2} + I_{3} \\ \end{aligned}$$

6 $$m_{22} = l_{2}^{2} \left( {0.25m_{2} + m_{3} } \right) + 0.25l_{3}^{2} m_{3} + l_{2} l_{3} m_{3} \; cos\left( {\theta_{3} } \right) + I_{2} + I_{3}$$

7 $$m_{23} = 0.25l_{3}^{2} m_{3} + 0.5l_{2} l_{3} m_{3} \; cos\left( {\theta_{3} } \right) + I_{3}$$

8 $$m_{31} = 0.25l_{3}^{2} m_{3} + 0.5l_{2} l_{3} m_{3} \; cos\left( {\theta_{3} } \right) + 0.5l_{1} l_{3} m_{3} \; cos\left( {\theta_{2} + \theta_{3} } \right) + I_{3}$$

9 $$m_{32} = 0.25l_{3}^{2} m_{3} + 0.5l_{2} l_{3} m_{3} \; cos\left( {\theta_{3} } \right) + I_{3}$$

10 $$m_{33} = 0.25l_{3}^{2} m_{3} + I_{3}$$

11 $$\begin{aligned} C_{1} \left( {\theta ,\dot{\theta }} \right) & = \left( {\dot{\theta }_{2} } \right)^{2} \left( { - 0.5l_{1} l_{2} m_{2} \; sin\left( {\theta_{2} } \right) - l_{1} l_{2} m_{3} \; sin\left( {\theta_{2} } \right) - 0.5l_{1} l_{3} m_{3} \; sin\left( {\theta_{2} + \theta_{3} } \right) } \right) \\ & \;\;\; - (\dot{\theta }_{3} )^{2} \left( {0.5l_{2} l_{3} m_{3} \; sin\left( {\theta_{3} } \right) - 0.5l_{1} l_{3} m_{3} \; sin\left( {\theta_{2} + \theta_{3} } \right)} \right) \\ & \;\;\; + \dot{\theta }_{1} \dot{\theta }_{2} \left( { - l_{1} l_{2} m_{2} \; sin\left( {\theta_{2} } \right) - 2l_{1} l_{2} m_{3} \; sin\left( {\theta_{2} } \right) - l_{1} l_{3} m_{3} \; sin\left( {\theta_{2} + \theta_{3} } \right)} \right) \\ & \;\;\; + \dot{\theta }_{2} \dot{\theta }_{3} \left( { - l_{1} l_{3} m_{3} \; sin\left( {\theta_{2} + \theta_{3} } \right) - l_{2} l_{3} m_{3} \; sin\left( {\theta_{3} } \right)} \right) \\ & \;\;\; + \dot{\theta }_{1} \dot{\theta }_{3} \left( { - l_{2} l_{3} m_{3} \; sin\left( {\theta_{3} } \right) - l_{1} l_{3} m_{3} \; sin\left( {\theta_{2} + \theta_{3} } \right)} \right) \\ \end{aligned}$$

12 $$\begin{aligned} C_{2} \left( {\theta ,\dot{\theta }} \right) & = \left( {\dot{\theta }_{1} } \right)^{2} \left( {0.5l_{1} l_{2} m_{2} \; sin\left( {\theta_{2} } \right) + l_{1} l_{2} m_{3} \; sin\left( {\theta_{2} } \right) + 0.5l_{1} l_{3} m_{3} \; sin\left( {\theta_{2} + \theta_{3} } \right) } \right) \\ & \;\; - 0.5(\dot{\theta }_{3} )^{2} \left( {l_{2} l_{3} m_{3} \; sin\left( {\theta_{3} } \right)} \right) - \dot{\theta }_{1} \dot{\theta }_{3} \left( { l_{2} l_{3} m_{3} \; sin\left( {\theta_{3} } \right)} \right) - \dot{\theta }_{2} \dot{\theta }_{3} \left( {l_{2} l_{3} m_{3} \; sin\left( {\theta_{3} } \right)} \right) \\ \end{aligned}$$

13 $$\begin{aligned} C_{3} \left( {\theta ,\dot{\theta }} \right) & = \left( {\dot{\theta }_{1} } \right)^{2} \left( {0.5l_{2} l_{3} m_{3} \; sin\left( {\theta_{3} } \right) + 0.5l_{1} l_{3} m_{3} \; sin\left( {\theta_{2} + \theta_{3} } \right)} \right) + 0.5\left( {\dot{\theta }_{2} } \right)^{2} \left( {l_{2} l_{3} m_{3} \; sin\left( {\theta_{3} } \right)} \right) \\ & \;\;\; + \dot{\theta }_{1} \dot{\theta }_{2} \left( {l_{2} l_{3} m_{3} \; sin\left( {\theta_{3} } \right)} \right) \\ \end{aligned}$$

14 $$\begin{aligned} G_{1} \left( \theta \right) & = m_{3} g\left( {l_{1} \; cos\left( {\theta_{1} } \right) + 0.5l_{3} \; cos\left( {\theta_{1} + \theta_{2} + \theta_{3} } \right) + l_{2} \; cos\left( {\theta_{1} + \theta_{2} } \right)} \right) \\ & \;\;\; + m_{2} g\left( {l_{1} \; cos\left( {\theta_{1} } \right) + 0.5l_{2} \; cos\left( {\theta_{1} + \theta_{2} } \right)} \right) + 0.5m_{1} gl_{1} \; cos\left( {\theta_{1} } \right) \\ \end{aligned}$$

15 $$G_{2} \left( \theta \right) = m_{3} g\left( {0.5l_{3} \; cos\left( {\theta_{1} + \theta_{2} + \theta_{3} } \right) + l_{2} \; cos\left( {\theta_{1} + \theta_{2} } \right)} \right) + 0.5m_{2} g l_{2} \; cos\left( {\theta_{1} + \theta_{2} } \right)$$

16 $$G_{3} \left( \theta \right) = 0.5m_{3} gl_{3} \; cos\left( {\theta_{1} + \theta_{2} + \theta_{3} } \right)$$

### Simscape multibody model

Within the Simulink® environment, Simscape™ enables the quick construction of physical model systems. Simscape allows for the creation of physical component models that are based on physical connections and easily interact with block diagrams and other modelling paradigms. Simscape add-on products give users access to more sophisticated parts and analysis tools. Simscape aids in the creation of control systems and the testing of system performance. Using MATLAB variables and expressions, we can parameterize our models, and Simulink can be used to create the control schemes for our physical system.

**Figure 2:**
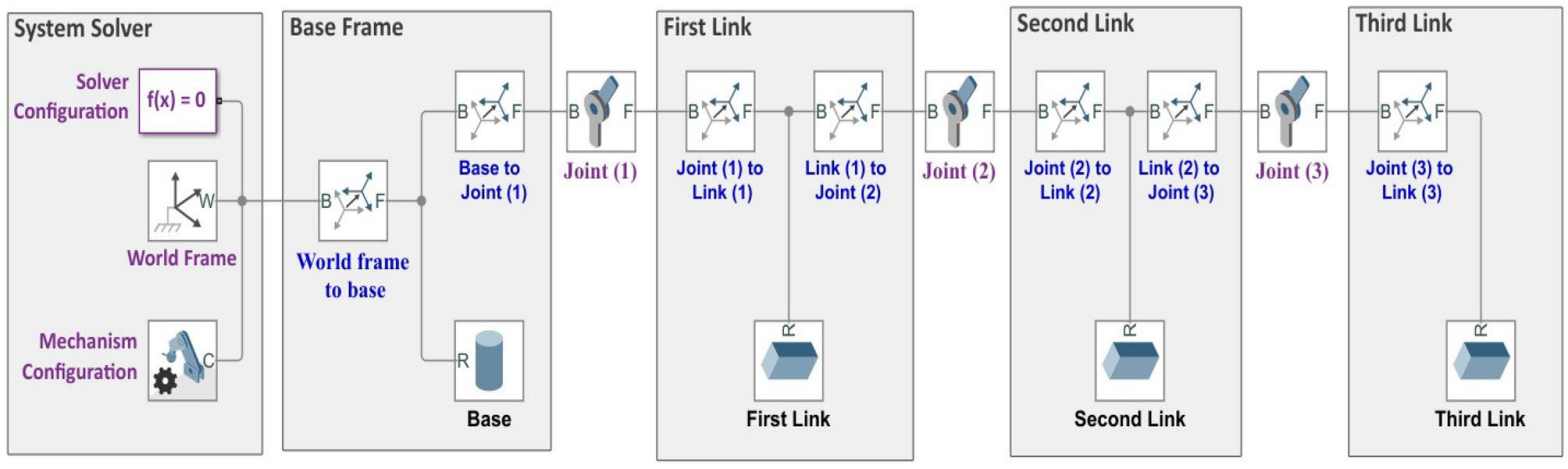
Simscape multibody model of the 3-DOF robotic maneuver.

The Simscape model, which represents the entire rigid link maneuver (RLM) system is shown in Fig. 2. All the physical parameters of the components that are generated with the MATLAB Simscape toolbox are listed in Table 1.

**Table 1:** The physical parameters of the links.

Parameters	Link 1	Link 2	Link 3
Mass	1 kg	1 kg	1 kg
I_zz_	0.0033 kg m^2^	0.0033 kg m^2^	0.0033 kg m^2^
Length	0.2 m	0.2 m	0.2 m
Cross-section area	$2.2 \times 2.2 \;{\text{cm}}^{{2}}$	$2.2 \times 2.2 \;{\text{cm}}^{2}$	$2.2 \times 2.2\; {\text{cm}}^{{2}}$

**Figure 3:**
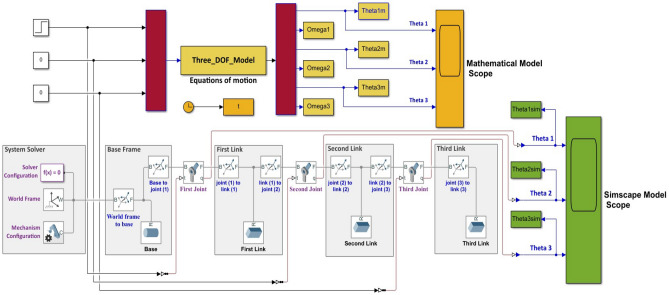
Step input signal applied to the first joint of the mathematical and Simscape models.

For the purpose of validating the mathematical model, a comparison between the open loop performance of the mathematical model and the Simscape model is developed when a step input signal is applied to the first joint as shown in Fig. 3.

**Figure 4:**
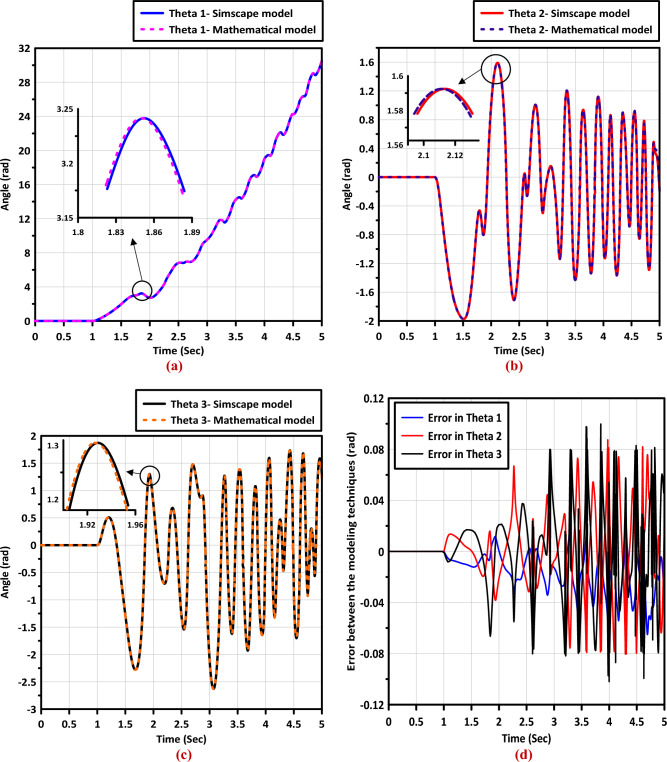
The response of both the mathematical and the Simscape models for: (**a**) the first link, (**b**) the second link, (**c**) the third link, and (**d**) the error in angles between the Simscape and mathematical models.

The response of the mathematical model compared with the response of the Simscape model is shown in Fig. 4. It is clearly obvious that the two model are identical, hence we can use the Simscape model in the remaining of this study and utilize its great facilities.

While our study focused on a 3 DOF system, the proposed model can be effectively applied to other DOF systems as well. The methodology employed in our approach can be extended to systems with higher degrees of freedom, with appropriate modifications and considerations for the increased complexity. However, it is important to note that the applicability of the proposed model to different DOF systems may depend on various factors, such as system dynamics, mechanical constraints, and control objectives. Therefore, further investigation and adaptation of the model may be necessary to ensure its effectiveness in specific DOF configurations.

## Sliding mode controller with PID surface

Before designing the sliding mode controller, it is essential to determine the appropriate sliding surface function. Subsequently, an equivalent controller and switching mode controller need to be constructed based on the system's states. The fundamental principle underlying this type of control is to ensure that the system's representative point, regardless of its initial conditions, resides on a hypersurface within the phase space. This hypersurface represents a collection of static relationships among the state variables, allowing for robust control of the system’’s evolution. There are two components in the sliding mode control as described in Eq. (17).

17 $${\varvec{\tau}}\left( {\varvec{t}} \right) = {\varvec{\tau}}_{{{\varvec{eq}}}} \left( {\varvec{t}} \right) + {\varvec{\tau}}_{{{\varvec{sm}}}} \left( {\varvec{t}} \right)$$

where ${\varvec{\tau}}_{{{\varvec{eq}}}} \left( {\varvec{t}} \right)$ is the continuous component and called equivalent controller and ${\varvec{\tau}}_{{{\varvec{sm}}}} \left( {\varvec{t}} \right)$ is the discontinuous component called switching mode controller.

The PID sliding surface for the SMC of each link can be specified with Eq. (18).

18 $${\varvec{s}}\left( {\varvec{t}} \right) = K_{p} {\varvec{e}}\left( {\varvec{t}} \right) + K_{I} \mathop \smallint \limits_{0}^{t} {\varvec{e}}\left( {\varvec{t}} \right)dt + K_{d} \dot{\varvec{e}}\left( {\varvec{t}} \right)$$

The diagonal matrices $K_{p} ,K_{I} \;{\text{and }}\;K_{d}$ represent the proportional, integral, and derivative gains of the PID surface, respectively. Additionally, the error signal ${\varvec{e}}\left( {\varvec{t}} \right)$ is defined as the difference between the desired angular position ${\varvec{\theta}}_{{\varvec{d}}} \left( {\varvec{t}} \right)$ and the measured angular position ${\varvec{\theta}}\left( {\varvec{t}} \right)$. Similarly, the derivative of the error signal $\dot{\varvec{e}}\left( {\varvec{t}} \right)$ is obtained by subtracting the measured angular speed $\dot{\varvec{\theta }}\left( {\varvec{t}} \right)$ from the desired angular speed $\dot{\varvec{\theta }}_{{\varvec{d}}} \left( {\varvec{t}} \right)$. These variables, namely ${\varvec{\theta}}_{{\varvec{d}}} \left( {\varvec{t}} \right)$, ${\varvec{\theta}}\left( {\varvec{t}} \right)$, $\dot{\varvec{\theta }}_{{\varvec{d}}} \left( {\varvec{t}} \right)$, and $\dot{\varvec{\theta }}\left( {\varvec{t}} \right)$, correspond to the vectors of desired angular position, measured angular position, desired angular speed, and measured angular speed of the robotic maneuver, respectively.

To calculate the continuous equivalent controller ${\varvec{\tau}}_{{{\varvec{eq}}}} \left( {\varvec{t}} \right)$ it is necessary that $\dot{\varvec{s}}\left( {\varvec{t}} \right) = 0$, so that we have the following:

19 $$\begin{aligned} K_{p} \varvec{\dot{e}}\left( \varvec{t} \right) & + K_{d} \varvec{\ddot{e}}\left( \varvec{t} \right) + K_{I} \varvec{e}\left( \varvec{t} \right) = 0~ \\ ~~\varvec{\ddot{e}}\left( \varvec{t} \right) & = - K_{d}^{{ - 1}} \left( {K_{p} \varvec{\dot{e}}\left( \varvec{t} \right) + K_{I} \varvec{e}\left( \varvec{t} \right)} \right) = \varvec{\ddot{\theta }}_{\varvec{d}} \left( \varvec{t} \right) - \varvec{\ddot{\theta }}\left( \varvec{t} \right) \\ \varvec{\ddot{\theta }}\left( \varvec{t} \right) & = \varvec{\ddot{\theta }}_{\varvec{d}} \left( \varvec{t} \right) + K_{d}^{{ - 1}} \left( {K_{p} \varvec{\dot{e}}\left( \varvec{t} \right) + K_{I} \varvec{e}\left( \varvec{t} \right)} \right) = M\left( \theta \right)^{{ - 1}} \left( {\varvec{\tau }_{{\varvec{eq}}} \left( \varvec{t} \right) - \varvec{C}\left( {\varvec{\theta },\varvec{\dot{\theta }}} \right) - \varvec{G}\left( \varvec{\theta } \right)} \right) \\ \varvec{\tau }_{{\varvec{eq}}} \left( \varvec{t} \right) & = M\left( \theta \right)\left( {\varvec{\ddot{\theta }}_{\varvec{d}} \left( \varvec{t} \right) + K_{d}^{{ - 1}} \left( {K_{p} \varvec{\dot{e}}\left( \varvec{t} \right) + K_{I} \varvec{e}\left( \varvec{t} \right)} \right)} \right) + \varvec{C}\left( {\varvec{\theta },\varvec{\dot{\theta }}} \right) + \varvec{G}\left( \varvec{\theta } \right)~ \\ \end{aligned}$$

The switching mode control law ${\varvec{\tau}}_{{{\varvec{sm}}}} \left( {\varvec{t}} \right)$ is chosen to be of the following form:

20 $${\varvec{\tau}}_{{{\varvec{sm}}}} \left( {\varvec{t}} \right) = M\left( \theta \right)\lambda {\text{sign}}\left( {{\varvec{s}}\left( {\varvec{t}} \right)} \right)$$

where $\lambda$ is a diagonal matrix of positive elements $\lambda_{ii}$.

The Lyapunov function is defined as following to construct the discontinuous switching mode controller and ensure the stability of the system^35^.

21 $$V = \frac{1}{2}{\varvec{s}}^{{\varvec{T}}} \left( {\varvec{t}} \right){\varvec{s}}\left( {\varvec{t}} \right)$$

By differentiating the Lyapunov function with respect to time along the trajectory of the system and utilizing Eqs. (19) and (20), the following expression is obtained.

22 $$\begin{aligned} ~\dot{V} & = \varvec{s}^{\varvec{T}} \left( \varvec{t} \right)\varvec{\dot{s}}\left( \varvec{t} \right) = \varvec{s}^{\varvec{T}} \left( \varvec{t} \right)\left( {K_{p} \varvec{\dot{e}}\left( \varvec{t} \right) + K_{d} \varvec{\ddot{e}}\left( \varvec{t} \right) + K_{I} \varvec{e}\left( \varvec{t} \right)} \right)~~ \\ & = \varvec{s}^{\varvec{T}} \left( \varvec{t} \right)\left( {K_{p} \varvec{\dot{e}}\left( \varvec{t} \right) + K_{I} \varvec{e}\left( \varvec{t} \right) + K_{d} \left( {\varvec{\ddot{\theta }}_{\varvec{d}} \left( \varvec{t} \right) - M\left( \theta \right)^{{ - 1}} \left( {\varvec{\tau }_{{\varvec{eq}}} \left( \varvec{t} \right) + \varvec{\tau }_{{\varvec{sm}}} \left( \varvec{t} \right) - \varvec{C}\left( {\varvec{\theta },\varvec{\dot{\theta }}} \right) - \varvec{G}\left( \varvec{\theta } \right)} \right)} \right)} \right)~ \\ & = - \varvec{s}^{\varvec{T}} \left( \varvec{t} \right)\varvec{~}K_{d} \lambda {\text{sign}}\left( {\varvec{s}\left( \varvec{t} \right)} \right)~ \\ & \le - \lambda_{m} \left\{ {K_{d} \lambda {\varvec{s}}\left( t \right)} \right\} \end{aligned}$$

where $\lambda_{m} \left\{ \cdot \right\}$ is the minimum eigen value of the product $K_{d} \lambda$.

According to the last equation, the system trajectories will be pushed toward the sliding surface in finite time^36^. Since, the signum function causes chattering, we will replace it with the saturated vector function ${\varvec{tanh}}\left( {{\varvec{s}}\left( {\varvec{t}} \right)} \right)$. Therefore, the new control law will be written as following:

23 $${\varvec{\tau}}_{{{\varvec{sm}}}} \left( {\varvec{t}} \right) = M\left( \theta \right)\lambda \tanh \left( {\frac{{{\varvec{s}}\left( {\varvec{t}} \right)}}{\delta }} \right)$$

where $\delta$ is a small positive gain, in this study $\delta$ is chosen to be $0.1$.

Finally, the total control law is given by the following equation:

24 $${\varvec{\tau}}\left( {\varvec{t}} \right) = M\left( \theta \right)\left( {\varvec{\ddot{\theta }}_{{\varvec{d}}} \left( {\varvec{t}} \right) + K_{d}^{ - 1} \left( {K_{p} \dot{\varvec{e}}\left( {\varvec{t}} \right) + K_{I} {\varvec{e}}\left( {\varvec{t}} \right)} \right) + \lambda \tanh \left( {\frac{{{\varvec{s}}\left( {\varvec{t}} \right)}}{\delta }} \right)} \right) + {\varvec{C}}\left( {{\varvec{\theta}},\dot{\varvec{\theta }}} \right) + {\varvec{G}}\left( {\varvec{\theta}} \right)$$

The elements of the diagonal matrices $k_{{p_{ii} }}$, $k_{{I_{ii} }}$, $k_{{d_{ii}}}$ and $\lambda_{ii}$ are the parameters to be tuned with the metaheuristic optimization algorithms.

## Metaheuristic optimization algorithm

Three distinct bees make up the honeybee colony in the bee colony: employed bees, onlooker bees, and scouter bees. Each kind of bee is responsible for a particular duty. The employed bees might be regarded of as playing an exploratory role because they are in charge of searching for nutrient-rich food sources over the entire search space. It should be noted that the number of food sources in the search region equals the total number of bees employed. The employed bees will share nectar information with the onlooker bees at the hive when they have finished their search in order to decrease the time spent in searching for new food sources in the following generation. The shared information comprises the richness of the nectar and the distance traversed to reach the food source. Each position of the food source represents a potential solution to the optimization problem, with the nectar's richness indicating its fitness value. Leveraging the acquired knowledge, the onlooker bees aim to identify new, high-quality food sources in the vicinity of selected sources. If a food supply has a high fitness value, it is more likely to be chosen. The onlookers play the exploitation rule, and there are exactly as many onlookers as there are employed bees, to guarantee the diversity of the solution space and prevent the algorithm from becoming stuck at a local optimum, scouter bees are added to each generation. If a food source cannot be improved after a certain number of trials, it is abandoned. The employed bee associated with the abandoned solution will then transform into a scouter bee and start scanning the entire search region for a new food source^21,23,26–28^.

The following subsections describe the five techniques that make up the detailed procedures of the enhanced artificial bee colony with multi-elite guidance (MGABC)^28^. The pseudocode of the MGABC optimization algorithm is described in Algorithm (1).

### Population Initialization

The algorithm initiates by stochastically generating food source sites ($SN$), which symbolize potential solutions in the search space. These sites can be perceived as the environment of the hive, where the search for solutions takes place. Within the range of the lower and upper boundaries of the parameters, the initial food sources $X_{i} = \left( {x_{i,1} , x_{i,2} , \ldots , x_{i,k} } \right)$ are produced randomly using Eq. (25)^20,21^, where $i \in \left( {1,2, \ldots ,SN} \right)$, $k \in \left( {1,2, \ldots ,S} \right)$ and $S$ denotes the dimension size of the parameters to be optimized.

25 $$x_{i,k} = x_{k}^{min} + rand\left( {0,1} \right) \times \left( {x_{k}^{max} - x_{k}^{min} } \right)$$

where $x_{k}^{min}$ and $x_{k}^{max}$ are the lower and upper bounds of the $Sth$ dimensions, respectively.

### Exploration technique

The solution search equation for employed bees should prioritize robust exploration, as their role is to discover new solutions throughout the entire search space. The enhanced solution exploratory equation described in Eq. (26) is therefore utilised by the exploratory bees, which comes from the CABC method proposed by Gao^37^.

26 $$v_{i,k} = x_{{r_{1} ,k}} + \phi_{i,k} \times \left( {x_{{r_{1} ,k}} - x_{{r_{2} ,k}} } \right)$$

where $X_{r1}$ and $X_{r2}$ are two distinct food sources that were randomly chosen from the initial population and are both different from $X_{k}$, in the range of $\left[ { - \;1,1} \right]$, $\phi_{i,k}$ is a uniformly distributed random number^21,23,26^.

### Exploitation technique

The onlooker bees should focus on exploitation rather than exploration because it is their duty to undergo comprehensive searches for possible promising food sources in the area in order to generate new offspring. As a result, Eq. (27)^28^ lists the new solution search method based on multiple elite solutions for the onlooker bees.

27 $$v_{i,k} = \left\{ {\begin{array}{*{20}c} {x_{e,k} + \phi_{i,k} \times \left( {x_{e,k} - x_{i,k} } \right)} & {if \; rand\left( {0,1} \right) \le MR} \\ {x_{i,k} } & {otherwise} \\ \end{array} } \right\}$$

where $X_{e} = \left( {x_{1,k} ,x_{2,k} , \ldots ,x_{{G_{e} ,k}} } \right)$ is one of the most promising elite solutions from the current population that was selected at random from the elite group. $Ge = q.SN$ denotes the size of the elite group. The control parameter $MR$, as employed by Akay and Karaboga^38^, serves the purpose of regulating the number of dimensions that can be transferred from the superior solution $X_{e}$ to the new solution $V_{i}$.

### Scouting technique

In each cycle, the algorithm checks to see whether there is any exhausted source that may be abandoned once all employed bees and onlooker bees have finished their searches. The counters that were updated throughout search are utilised to determine whether a source should be abandoned^23,37^. If the value of the counter surpasses the predefined “limit” the source associated with this counter is considered depleted and abandoned within the context of the ABC algorithm^21,23,36^. If ${X}_{i}$ represents the abandoned food source, then the scout bee generates a new random food source according to Eq. (25).

### Enhanced neighborhood search operator technique

The modified operator that performs additional exploration and aids in locating better alternatives or even the best solution, if one solution is unfortunate became trapped by one of the local optima is listed in Eq. (28)^22,23,27,28,38^

28 $$TX_{i} = r_{1} \cdot X_{i} + r_{2} \cdot X_{e1} + r_{3} \cdot \left( {X_{e2} - X_{e3} } \right)$$

where $X_{e1}$, $X_{e2}$, and $X_{e3}$ are three food sources chosen at random from the elite group, and they must be distinct from $X_{i}$. Table 2 lists the various MGABC design parameters design parameters that are utilised to maximize controller gains.

**Table 2:** The internal design parameters of MGABC optimization algorithm.

DP. No	The MGABC	
	Algorithm parameter	Value
1	Colony size, $SN$	20
2	Iterations, $T$	50
3	Modification rate, $MR$	0.5
4	Size of elite group,$Ge$	3

## Controller optimization for trajectory tracking

**Figure 5:**
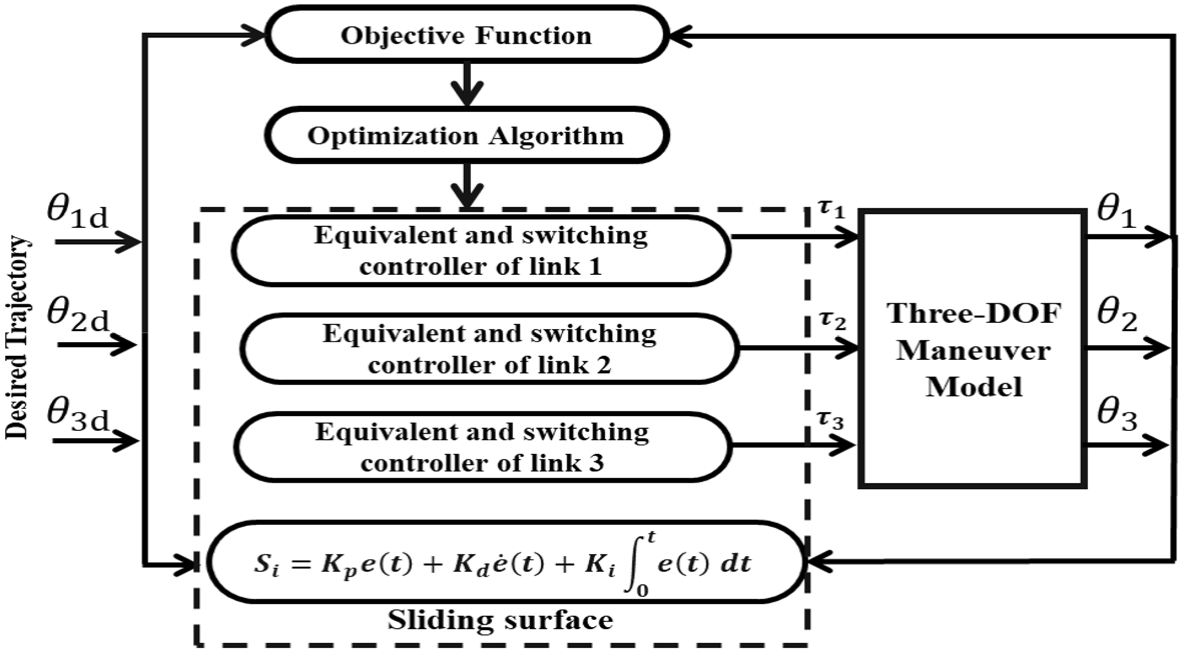
Schematic diagram of the tuning process.

All of the simulations provided in this study were run in MATLAB/SIMULINK with the ODE45 solver using 0.001 s sampling time and the simulation time is taken to be 5 s on a personal computer with an Intel® Core™ i7-10750H CPU running at 2.60 GHz, 16 GB of RAM, and a 64-bit Win 11 operating system. Equations (29)–(31) list the desired trajectories. Figure 5 illustrates a schematic diagram depicting the fundamental tuning scheme of the SMC-PID control method.

Convergence of results is a key aspect in any optimization process. While there is no fixed iteration count to guarantee an optimal solution, we monitored the convergence behavior of the objective function values. Once we observed negligible deviations and attained a near-optimal solution, we decided to terminate the iterations. Furthermore, it is worth mentioning that each run of the optimization process yields global optimal solutions, although with varying iterations number. We aimed to assess the performance of various optimization algorithms for our particular problem, considering that the most efficient algorithm reported in previous publications may not necessarily be the most suitable for our specific problem domain. To ensure a comprehensive evaluation, we explored multiple optimization algorithms with different parameter settings, including the number of iterations.

In our work, we conducted experiments with different iteration values, specifically starting with 30, 40, 50 and 60 iterations. Through careful analysis, we observed that after 50 iterations, the differences in the objective function values became negligible, indicating that the optimal value was reached.

29 $$\theta_{d1} = \sin \left( {2t} \right) - 0.1$$

30 $$\theta_{d2} = \cos \left( {2t} \right) - 0.1$$

31 $$\theta_{d3} = \cos \left( {2t} \right) - 0.1$$

**Figure 6:**
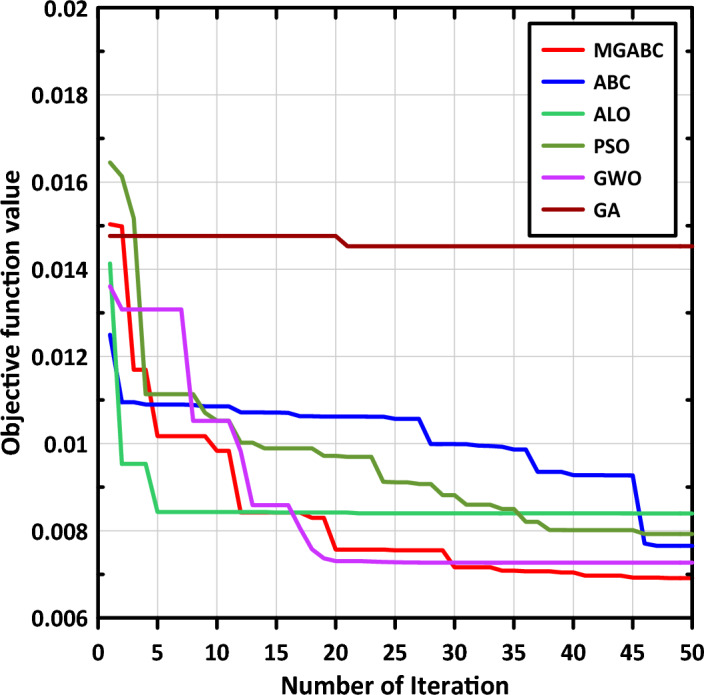
Objective function versus iteration curves.


The integral time absolute error (ITAE) listed in Eq. (32) served as the objective function (OBJF) of the tuning process. The curves of OBJF versus iteration for MGABC and other optimization techniques from literature study such as Particle Swarm Optimization (PSO), Genetic Algorithm (GA), Artificial Bee Colony (ABC), Ant Lion Optimizer (ALO) and Grey Wolf Optimizer (GWO) are depicted in Fig. 6. Additionally, it should be highlighted that optimal controlling parameters remained unaltered throughout the studies.

32 $$ITAE = \mathop \smallint \limits_{0}^{t} t\left| {{\varvec{e}}\left( {\varvec{t}} \right)} \right|dt$$

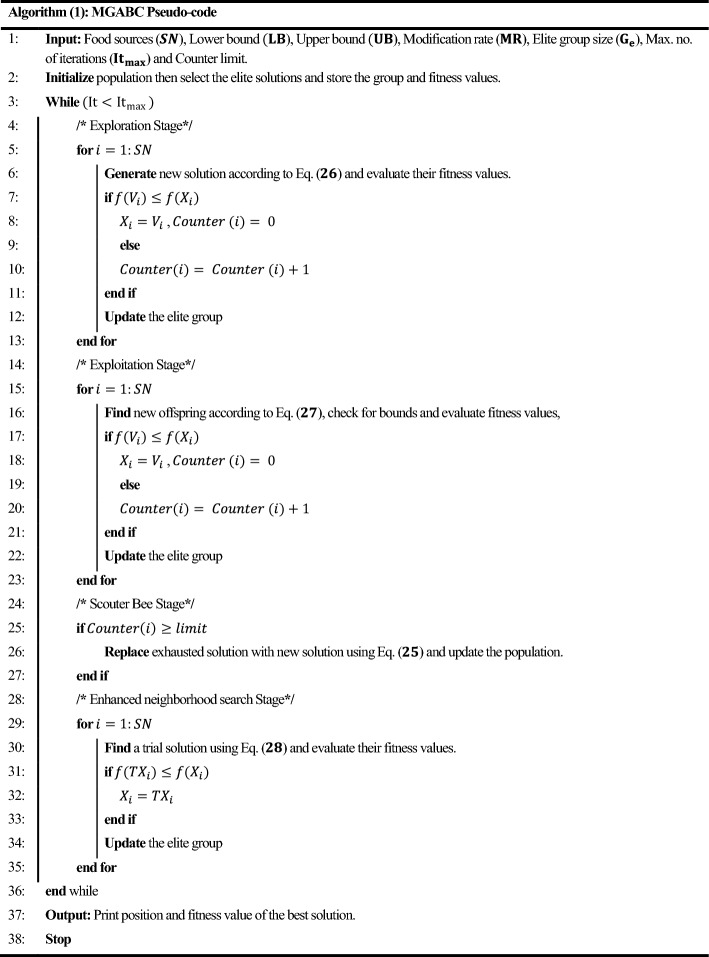


Table 3 displays the parameters of the robotic system model’s upper and lower search spaces, together with the MGABC optimizer’s tuned values. The lower bound values for the sliding surface and switching mode gain in the controller were specifically chosen to ensure the stability of Eq. (18). The process of selecting these values incorporated several factors, including the physical properties of the system such as inertia and weight of each link, as well as the system dynamics, desired performance, and stability criteria. These considerations were crucial in determining the lower bounds that would guarantee the stability of the system and ensure its proper functioning.

**Table 3:** The optimum parameter values of 3-DOF system model solved based on MGABC optimizer.

Parameter	Lower bound			Upper bound			Optimum		
	Link 1	Link 2	Link 3	Link 1	Link 2	Link 3	Link 1	Link 2	Link 3
${k}_{p}$	5	5	5	50	50	50	50	50	50
${k}_{I}$	0.1	0.1	0.1	10	10	10	9.973	10	9.330
${k}_{d}$	0.1	0.1	0.1	1	1	1	0.206	0.100	0.109
$\lambda$	50	50	50	300	300	300	300	295.986	299.837

While the upper bound values were selected in a more approximate manner. While these values were not derived through a rigorous optimization process, they were carefully chosen to establish a reasonable range for the parameters without violating any operational constraints. The intent behind this selection was to encompass a broad range of feasible values that could be explored for experimentation and analysis purposes.

**Figure 7:**
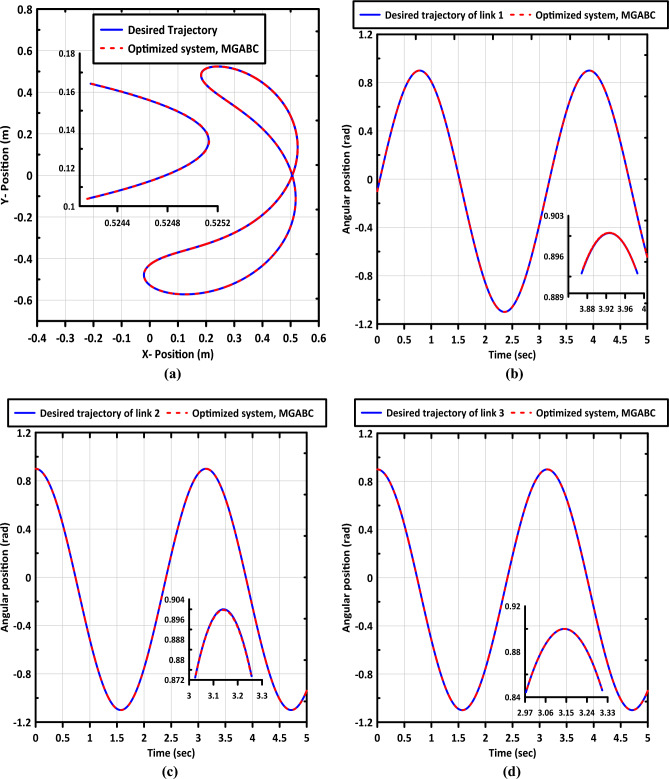
Shows (**a**) $\mathrm{X}-\mathrm{Y}$ plot of end-effector and trajectory tracking curves of (**b**) link (1), (**c**) link (2), and (**d**) link (3).

Table 4 presents the minimum, average, and maximum OBJFs obtained by the MGABC optimizer in comparison to other meta-heuristic algorithms in the literature where 10 separate runs of each algorithm were performed. It is notable that the MGABC algorithm produced the minimum results overall runs with the lowest standard deviation with the lowest OBJF values. Figure 7a shows the $X - Y$ plot of end-effector and Fig. 7b–d demonstrate the trajectory tracking of each link. As we can see the desired and actual trajectories are nearly identical.

**Table 4:** The optimum OBJF of the MGABC algorithm in comparison with other algorithms.

Algorithm	Minimum	Maximum	Mean	Standard deviation
ABC	0.007653312	0.00831256	0.008040319	0.000270904
PSO	0.007925914	0.008892364	0.008292557	0.000448724
GWO	0.007266579	0.010600297	0.008693191	0.00140179
ALO	0.008393979	0.00889129	0.008791828	0.000222404
GA	0.014525562	0.01920016	0.016967015	0.001764241
MGABC	0.006914077	0.007176508	0.007025913	0.00010948

**Figure 8:**
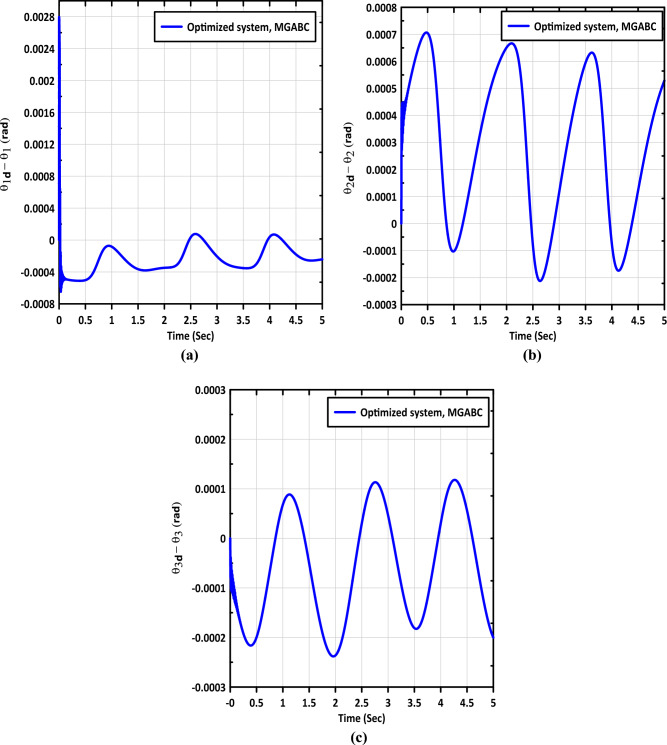
The error in signals of (**a**) link (1), (**b**) link (2), and (**c**) link (3).

Figure 8a–c shows the error in trajectories of links 1, 2 and 3, respectively. The controller exhibits superior tracking performance, as evidenced by the objective function (OBJF) values of links 1, 2, and 3. Which are $0.00215$, $0.00126$ and $0.00350$ respectively. That illustrates how the MGABC method outperforms other algorithms in the literature in terms of result superiority and robustness.

## Detailed simulated performance evaluation

In order to ensure robustness, a controller must effectively mitigate the impact of both measured and unmeasured noise and disturbance, as well as exhibit adaptability to uncertainties present in the system^3^. In this section, we extensively investigate the controller’s ability to reject disturbance and noise, while also evaluating its robustness against uncertainties in the payload’s mass. Furthermore, we examine the controller’s adaptability to the flexibility exhibited by the joints in the system.

### Robustness vs disturbance and noise

**Figure 9:**
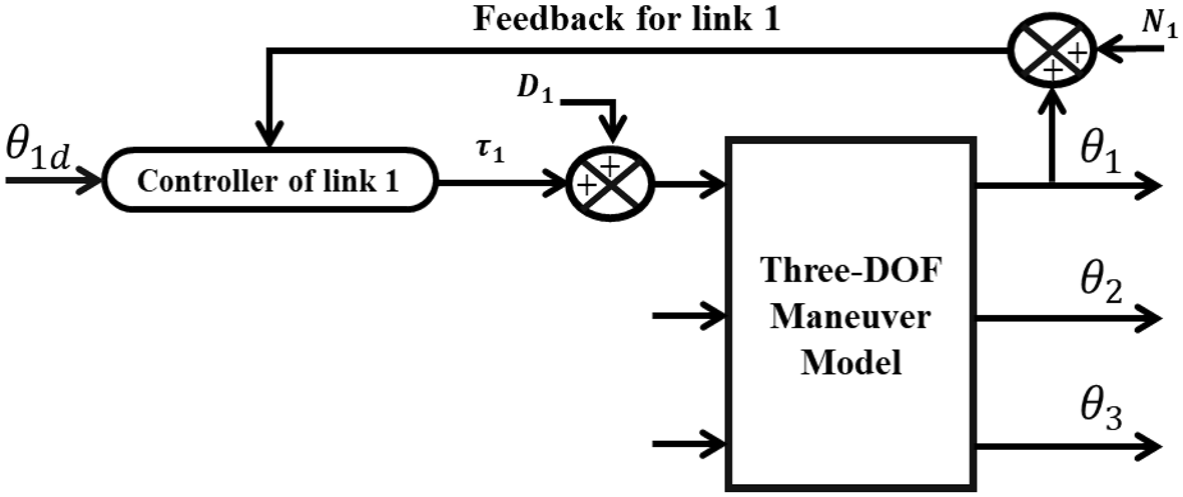
The robotic system in the presence of disturbance and noise.

The dynamic disturbance signal and noise, listed in Eqs. (33) and (34), are injected at the output of each controller and sensor of joint at the same time as shown in Fig. 9.

33 $$\tau_{i} = per \times rand \times \tau_{i}$$

34 $$N_{i} = per \times rand \times \left( {\theta_{{d_{i} }} + \theta_{i} } \right)$$

In the given context, the variable “$rand$” represents a randomly generated number within the inclusive range of $\left[ {0,1} \right]$. The symbols $\tau_{i}$, $\theta_{{d_{i} }}$ and $\theta_{i}$, where $i$ takes values of 1, 2, and 3, correspond to the applied torques, desired angles, and measured angles, respectively. The systems performance is checked for varying percentage $\left( {per} \right)$ values from $1\%$ to severe conditions of $5\%$ percentage of disturbance and noise. The corresponding OBJFs values of the optimized controller with MGABC to the variation in percentage of disturbance and noise are listed in Table 5.

**Table 5:** OBJFs values for variation in percentage of noise and disturbance.

Percentage	OBJFs values
	MGABC
$1\%$	0.00698
$2\%$	0.00715
$3\%$	0.00733
$4\%$	0.00761
$5\%$	0.00792

The controller is designed to minimize the objective function (OBJF), and its effectiveness is evident from the observed percentage increase in OBJF values. This increase ranges from $0.954\%$ under conditions of low disturbance and noise to $14.55\%$ under conditions of severe disturbance and noise. The controller's ability to mitigate the impact of disturbance and noise is demonstrated by the relatively small increase in OBJF values, indicating its robustness and effectiveness in maintaining desired performance even in challenging situations with significant disturbances and noise.

### Robustness vs the variation in mass of payload

The primary objective of a maneuvering system is to perform the task of gripping and manipulating objects with diverse masses using its end-effector. When there is a change in the mass of the end-effector, it introduces a new configuration to the system, necessitating the need for a robust controller. The robust controller is essential to mitigate the impact of variations in the end-effector mass and ensure stable and reliable performance^3^.

**Figure 10:**
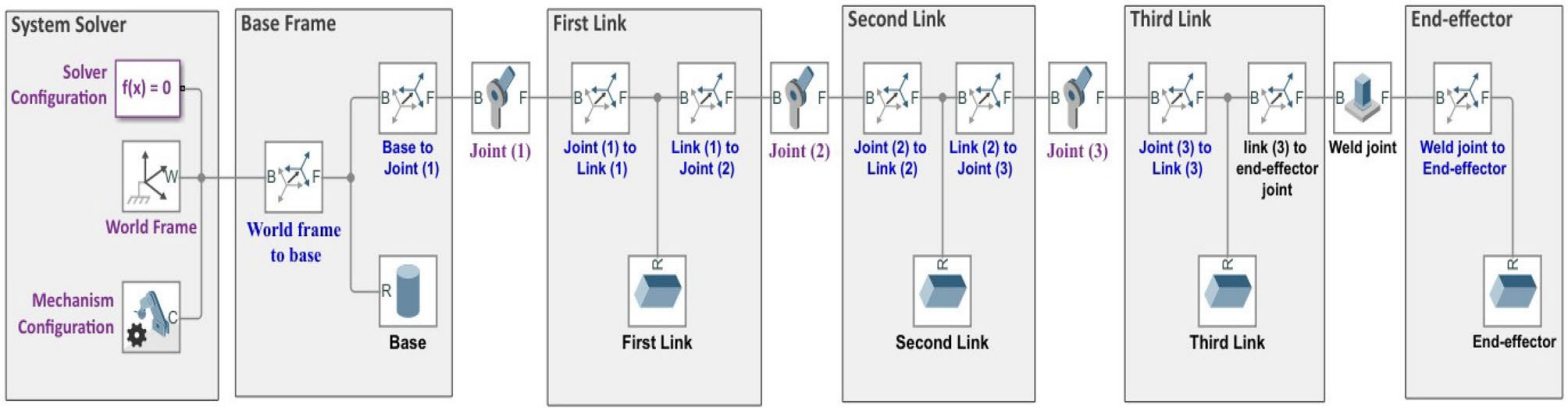
Simscape multibody model of the 3-DOF maneuver with end-effector.

In this study we utilized the great facilities of the Simscape multibody toolbox and easily added a new component which describes the end-effector tool as shown in Fig. 10. The variation in the mass of end-effector follows Eq. (35), and the corresponding OBJFs values of the optimized controller with MGABC to the variation in percentage of mass are listed in Table 6.

35 $$Uncertainty = per \times m$$

where $m$ is the mass of the link $i$ and in our study $m = 1\; {\text{Kg}}$ and the percentage $(per$) of uncertainty ranges from $10 \%$ to severe uncertainty conditions of $50 \%$ (i.e., from $0.1$ to $0.5\; {\text{Kg}}$).

**Table 6:** OBJFs values for variation in percentage of mass.

Percentage	OBJFs values
	MGABC
$10\%$	$0.00731$
$20\%$	$0.00749$
$30\%$	$0.00789$
$40\%$	$0.00802$
$50\%$	$0.00822$

The optimized controller utilizing the MGABC algorithm demonstrates resilience in the face of payload mass variations. Through analysis, it is observed that the OBJF values exhibit an inflation ranging from $5.726\%$ under low uncertainty conditions to $18.887\%$ under severe uncertainty conditions. This indicates that the controller maintains its performance and robustness even in challenging situations with significant uncertainties.

### Adaptability to joint flexibility

**Figure 11:**
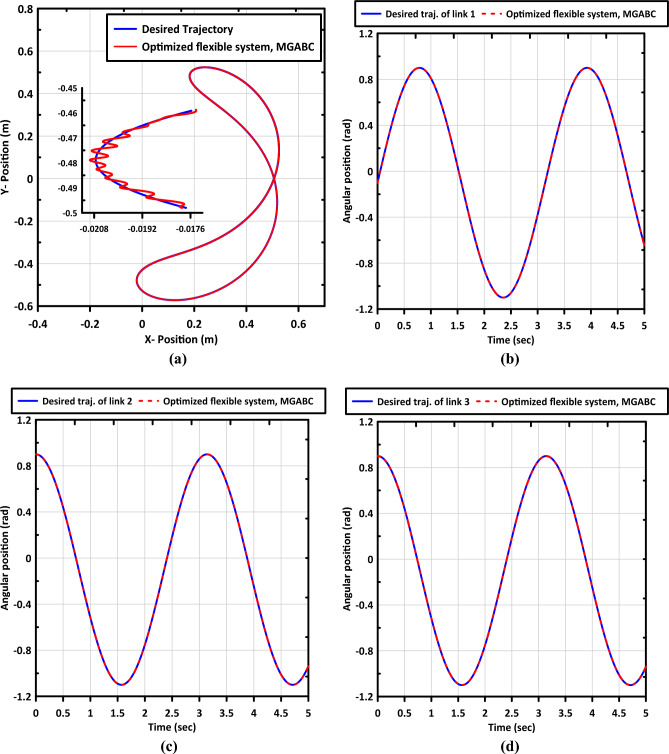
Shows (**a**) $\mathrm{X}-\mathrm{Y}$ plot of end-effector of the FLM and trajectory tracking curves of (**b**) link (1), (**c**) link (2), and (**d**) link (3).

Flexible link manipulators (FLMs) offer numerous advantages such as reduced material consumption, lower power requirements, lighter weight, fewer actuators, and improved maneuverability. Despite these benefits, the control of FLM systems presents several unresolved challenges that must be addressed before their widespread adoption in practical real-world applications^1,39^. In this section, we will investigate the capability of the optimized controller approach to handle the influence of joint flexibility. The Simscape multibody toolbox is employed to simulate the system, allowing us to model the three joints as flexible. The internal mechanics of these joints are characterized by a spring stiffness of $1$ and an equivalent viscous damping of $0.001$. This setup enables us to analyze the response of the controller in the presence of joint flexibility and evaluate its adaptability to such conditions. $X-Y$ plot of end-effector and trajectory tracking curves are shown in Fig. 11. The proposed controller shows superior tracking performance with little vibration in the movement of the end effector.

## Conclusion

This paper highlights the significance of utilizing the MATLAB Simscape Multibody Toolbox as a powerful tool for modeling and controlling both rigid and flexible maneuvers. The optimization of a sliding mode controller with a proportional integral derivative (PID) surface is achieved by employing an enhanced artificial bee colony algorithm with multi-elite guidance (MGABC). The selection of the optimal sliding surface poses a challenge due to the vast search space, leading many existing optimization algorithms to become trapped in local optima and fail to identify the optimal parameters for the sliding surface and switching mode controller gain. The MGABC algorithm is chosen for its exceptional exploratory and exploitative techniques, enabling it to find the best solution. The convergence analysis conducted in this study provides evidence of the MGABC algorithm's exceptional capability to overcome local minima through its proficient exploration and exploitation techniques. This algorithm demonstrates superiority over other algorithms mentioned in the existing literature in terms of its ability to navigate and escape from local minima solutions. The investigation focuses on disturbance and noise rejection, as well as robustness against uncertainty in payload mass. The proposed controller demonstrates the ability to mitigate the effects of disturbances and noises, maintaining the objective function (OBJF) at a minimum even in highly noisy and disturbed systems. The controller effectively minimizes the OBJF, with the percentage increase in OBJF values ranging from 0.954% under low disturbance and noise conditions to 14.55% under severe disturbance and noise conditions. Additionally, the optimized controller exhibits resilience to variations in payload mass analysis, with the percentage increase in OBJF values ranging from 5.726% under low uncertainty conditions to 18.887% under severe uncertainty conditions. Furthermore, the adaptability of the controller to joint flexibility is evaluated, demonstrating superior tracking performance with minimal vibration in the movement of the end effector.

## Data Availability

The datasets generated during and/or analyzed during the current study are available from the corresponding author on reasonable request.
